# The assessment of spiritual well-being in cancer patients with advanced disease: which are its meaningful dimensions?

**DOI:** 10.1186/s12904-020-0534-2

**Published:** 2020-03-06

**Authors:** Elisa Rabitti, Silvio Cavuto, Luca Iani, Simona Ottonelli, Francesco De Vincenzo, Massimo Costantini

**Affiliations:** 1 Psycho-Oncology Unit, Azienda USL-IRCCS di Reggio Emilia, Reggio Emilia, Italy; 2 SC Infrastruttura Ricerca e Statistica, Azienda USL-IRCCS di Reggio Emilia, Reggio Emilia, Italy; 3 Department of Human Sciences, European University of Rome, Rome, Italy; 4 Associazione Gigi Ghirotti – ONLUS, Genoa, Italy; 5 Azienda USL-IRCCS di Reggio Emilia, Reggio Emilia, Italy

**Keywords:** Spirituality, Cancer, Well-being

## Abstract

**Background:**

Spirituality is particularly important for patients suffering from life-threatening illness. Despite research showing the benefits of spiritual assessment and care for terminally ill patients, their spiritual needs are rarely addressed in clinical practice. This study examined the factor structure and reliability of the Functional Assessment of Chronic Illness Therapy-Spiritual (FACIT-Sp) in patients with advanced cancer. It also examined the clinical meaning and reference intervals of FACIT-Sp scores in cancer patients subgroups through a literature review.

**Methods:**

A forward-backward translation procedure was adopted to develop the Italian version of the FACIT-Sp, which was administered to 150 terminally ill cancer patients. Exploratory factor analysis was used for construct validity, while Cronbach’s α was used to assess the reliability of the scale.

**Results:**

This study replicates previous findings indicating that the FACIT-Sp distinguish well between features of meaning, peace, and faith. In addition, the internal consistency of the FACIT-Sp was acceptable. The literature review also showed that terminal cancer patients have the lowest scores on the Faith and Meaning subscales, whereas cancer survivors have the highest scores on Faith.

**Conclusions:**

The Italian version of the FACIT-Sp has good construct validity and acceptable reliability. Therefore, it can be used as a tool to assess spiritual well-being in Italian terminally ill cancer patients. This study provides reference intervals of FACIT-Sp scores in newly diagnosed cancer patients, cancer survivors, and terminally ill cancer patients and further highlights the clinical meaning of such detailed assessment.

## Background

Previous research has yielded supportive evidence on the positive influence of spiritual well-being in health care, especially in the context of a serious and life-limiting illness such as cancer [1]. It was shown to promote better psychosocial adjustment to cancer [2, 3] and cancer-related growth [4, 5]. There is a growing incidence of cancer worldwide that poses a considerable threat to quality of life and public health [6]. Therefore, it is essential to pay attention to patients’ spiritual needs [7]. Despite research showing the benefits of spiritual assessment and care for cancer patients, their spiritual needs are not supported by the medical system [8].

Spirituality may provide “a context in which people can make sense of their lives, and feel whole, hopeful and peaceful even in the midst of life’s most serious challenges” [9]. A more recent definition by Visser, Garssen and Vingerhoets [10] states that spirituality refers to “one’s striving for and experience of a connection with the essence of life of which the experiences of meaning in life and connectedness are central elements”. Spirituality is particularly relevant for patients suffering from life-threatening illness, especially at the end of life [11]. Indeed, these patients may struggle with questions about mortality or the meaning of life that they had not considered before they became ill. Although some patients may turn to religion to meet their existential needs, others find relief through non-religious spiritual beliefs.

According to the Biopsychosocial-Spiritual Model [12], spirituality is positively associated with Quality of Life (QOL) [9, 13, 14]. When spiritual needs are substantially unmet, end of life patients are forced to grapple with an overall burden of daily distress and worries that affect their emotional and spiritual well-being [15] as well as health care decision-making [8, 16, 17]. Nowadays, spirituality is recognized by palliative care specialists as an important strategy to cope with life-threatening illness.

An increasing number of researchers have investigated and included the assessment of spirituality in health care [18, 19]. Spiritual well-being is a component of spirituality [20] that can be defined as “a sense of meaning in life, harmony, peacefulness, and a sense of drawing strength and comfort from one’s faith” [21]. Perception of meaning in life refers to a sense of understanding, significance, and purpose in life [22]. Peace includes a sense of being reconciled to one’s adverse life circumstances [3]. Finally, faith is a sense of comfort or strength one derives from one’s faith and spiritual beliefs [23].

One of the most widely used instruments for measuring spiritual well-being in patients with chronic and/or life-threatening diseases is the Functional Assessment of Chronic Illness Therapy-Spiritual (FACIT-Sp) [23]. It was originally validated in USA with cancer and HIV/AIDS patients, demonstrating good psychometric properties [23]. A principal components analysis on the 12 items of the FACIT-Sp revealed two distinct factors that were related to Meaning/Peace and Faith. Given that meaning suggests a cognitive aspect of spirituality and peace an affective component, Canada and colleagues [21] used confirmatory factor analysis to compare the original two-factor model with the three-factor solution. The study of Canada and colleagues [21] supported a three-factor solution of the FACIT-Sp (Meaning, Peace, and Faith), which represented an improvement over the original version and also enabled a more detailed analysis of the contribution of different facets of spirituality on QOL. The clinical meaningfulness of the three-factor model was subsequently confirmed [3, 24]. Although it was mainly used in oncologic settings, the original instrument has been used also with different populations and settings [25–29].

To our knowledge, only one study [24] was conducted to examine the factorial validity of the FACIT-Sp with advanced cancer patients. However, these patients were newly diagnosed with advanced cancer. No previous study has investigated the factorial validity of FACIT-Sp in patients with advanced and terminally ill cancer who were also no newly diagnosed.

The aim of this study was twofold. First, to culturally adapt the Italian version of the FACIT-Sp in a sample of cancer patients and to examine its acceptability, factorial validity, and reliability. Second, to examine and interpret the clinical meaning of FACIT-Sp scores through a literature review of published articles and to define reference intervals for FACIT-Sp scores in cancer patients subgroups. Such reference intervals can be helpful to interpret the distribution of the related scores in the cited patients subgroups from a statistical perspective, laying the groundwork for further investigations to better clarify their clinical meaning.

## Methods

### Study design and procedure

This study is a secondary analysis of the Palliative Care Outcome Scale (POS) Italian validation study [30]. We also conducted a literature review to examine and interpret the clinical meaning of FACIT-Sp scores and to define reference intervals for FACIT-Sp scores for newly diagnosed cancer patients, cancer survivors, and terminal cancer patients.

The palliative care teams comprised of doctors, nurses, and psychologists who administered the questionnaires during staff meetings. Informed written consent was obtained from all participants before data collection, after being informed about the voluntary nature of participation, and the right to withdraw from the study at any moment. The study was approved by the Ethical Committee of the National Institute for Cancer Research of Genoa (Deliberation EC07.001 of 19 February 2007).

### Participants

The study was conducted with a sample of 150 advanced and terminally ill cancer patients attending various palliative care services (hospices or home care). Eligible patients had a diagnosis of cancer, were 18 years of age or more, and gave their consent to participate in the study.

### Measures

The English original version of the FACIT-Sp, officially provided by the FACIT.org group (www.facit.org), was translated into Italian using a forward-backward translation method to establish cross-language equivalence. The instrument includes 12 items that measure aspects of spiritual well-being related to meaning and purpose in life, peacefulness, and a sense of strength and comfort one derives from one’s faith and spiritual beliefs. Participants were required to indicate how true each statement was for them during the previous week on a 5-point scale, ranging from 0 (*not at all*) to 4 (*very much*). Higher scores indicate higher levels of spiritual well-being. This instrument takes around 5–10 min to complete. We used the ECOG Performance Status to measure how cancer impacts patients’ daily living abilities [31].

### Statistical analysis

We assessed the acceptability of the instrument to respondents through compliance (% of patients who completed the questionnaire) and adherence (% of patients who completed each item). We assumed that 5–10% was an acceptable proportion of missing for each item of the questionnaire, taking into account the settings where the FACIT-Sp was administered. The relationship between FACIT-Sp subscales and total scores and socio-demographic variables was evaluated using Pearson’s *r* and Spearman’s rho, whereas the internal consistency was assessed by Cronbach’s α. We also calculated Spearman’s rho of FACIT-Sp subscales and total scores with ECOG index. We used *t*-test to compare the FACIT-sp total scores between males and females.

For inclusion in the review, we considered all papers that reported mean and standard deviation for the FACIT-Sp scores. To define the lower and upper limits of the reference intervals for the FACIT-Sp scores, we classified all papers into three categories according to the patients’ characteristics:

1. newly diagnosed cancer patients;
2. cancer survivors;
3. terminal cancer patients.

The selection of categories was guided by the expectation that reference intervals would be different according to these patients’ characteristics. For each FACIT-Sp score we calculated weighted means and weighted standard deviations, where the weights are determined by the number of patients. To define the reference intervals, we assumed a normal distribution of scores, then we used the 2.5th percentile as the lower limit and the 97.5th percentile as the upper one.

### Literature review

An electronic search using Embase, Medline, Cochrane Library, Cinahl and Psycinfo from 2002 to June 2016 was performed to identify the literature on the FACIT-Sp scale. The search terms used were Functional Assessment of Chronic Illness Therapy-Spiritual, FACIT-Sp, FACIT, and the search limits used were adults (from 18 years), English, French, Italian, and Spanish languages. The inclusion criteria for the review were published studies with cancer patients in all stages of disease containing FACIT-Sp scores. Unpublished studies or proceedings from conferences were excluded from the review.

## Results

### Acceptability of the instrument and disease characteristics

One hundred thirty-six of the 150 patients completed the FACIT-Sp, with a compliance of 90.6%. The adherence to the instrument was high, with a range from 92% (item 12) to 100% (item 1 and 7).

Ninety-five per cent of patients had a solid cancer, of which most had gastrointestinal cancer (41.2%). The majority of patients had a performance status of 3 (limited self-care) or 4 (completely disabled). Additional demographic information and disease-related characteristics of the patients are provided in Table 1.

**Table 1:** Patients’ characteristics (*n* = 136)

	Mean (SD)	Range
**Age**	70.5 (12.8)	31–100
**Gender**	***n***	**%**
Male	69	50.7
Female	67	49.3
**Education**	***n***	**%**
Anyone / primary school	64	48.5
High school	34	25.8
College	26	19.7
Graduate	8	6.1
**Marital status**	***n***	**%**
Single	41	30.1
Married	82	66.7
Missing	13	9.5
**Centre type**	***n***	**%**
Hospice	62	45.6
Home care	74	54.4
**Cancer type**	***n***	**%**
Hematologic	6	4.4
Solid	129	95
NAS	1	0.7
**Performance status (ECOG)**	***n***	**%**
0	3	2.2
1	8	5.9
2	16	11.8
3	82	60.3
4	27	19.9

### Demographic and psychological variables

There were no differences between males and females in terms of FACIT-Sp total score (Males: *M* = 22.83 ± 7.05, Females: *M* = 22.86 ± 7.01, *t*(130) = − 0.025, *p* = .980). Moreover, the FACIT-Sp total score was not correlated with age (*r* = −.11), education (rho Spearman = .03), marital status (rho Spearman = .03), cancer type (rho Spearman = −.04), and the ECOG (rho Spearman = −.13).

### Exploratory factor analysis

We used an exploratory factor analysis with principal axis factoring extraction to examine the structure of the FACIT-Sp. The number of factors was determined by a parallel analysis using the SPSS syntax [32]. This criterion suggested a three-factor solution (eigenvalues 2.8, 3.0, 2.7), accounting for 55% of the variance. Previous research suggests that the scales are correlated [21]. Thus, we applied an oblimin rotation to this solution. Correlations among factors were similar to those in previous research, with Meaning and Peace factors correlating at .43, and Meaning and Faith as well as Peace and Faith correlating at .28. The first factor (34% of total variance) was defined by five items reflecting meaning; the second factor (13% of total variance) was defined by three items referring to faith; finally, the third factor (8% of total variance) was defined by four items reflecting peace. Each item loaded on only one of the three factors with a value greater than 0.40 (see Table 2). All but one of the four items that make up the Faith scale loaded on this factor. The one item on the Faith scale (“I know that whatever happens with my illness, things will be okay”) that did not load on this factor had a .55 loading on the Meaning scale. Table 2 reports the factor loadings of FACIT-Sp Scale items (the allocation of items to subscales according to different validation studies is provided in Table S1).

**Table 2:** Factor loadings of FACIT-Sp items following principal axis factor extraction with oblimin rotation

FACIT-Sp Italian item *(English)*	*Meaning*	*Faith*	*Peace*
5 Sento che la mia vita ha uno scopo (*I feel a sense of purpose in my life*)	**.88**	−.01	−.07
2 Ho delle buone ragioni per continuare a vivere (*I have a reason for living*)	**.74**	.00	.00
12 Sono certo/a che tutto andrà bene, indipendentemente dall’esito della mia malattia (*I know that whatever happens with my illness, things will be okay*)	**.55**	−.25	−.02
3 La mia vita è stata produttiva (*My life has been productive*)	**.47**	.03	.01
8 La mia vita manca di significato e scopo (*My life lacks meaning and purpose*)	**.41**	.12	.23
10 Trovo forza nella mia fede o nel mio credo spirituale (*I find strength in my faith or spiritual beliefs*)	−.03	**−.96**	.08
9 Trovo conforto nella mia fede o nel mio credo spirituale (*I find comfort in my faith or spiritual beliefs*)	.00	**−.91**	.06
11 La mia malattia ha rafforzato la mia fede o il mio credo spirituale (*My illness has strengthened my faith or spiritual beliefs*)	.03	**−.73**	−.03
7 Mi sento in armonia con me stesso/a (*I feel a sense of harmony within myself*)	.20	−.07	**.70**
1 Mi sento sereno/a (*I feel peaceful*)	−.02	−.01	**.68**
4 Ho difficoltà a trovare la tranquillità d’animo (*I have trouble feeling peace of mind*)	−.07	.04	**.68**
6 Sono in grado di trovare conforto dentro me stesso/a (*I am able to reach down deep into myself for comfort*)	.08	−.27	**.56**

### Reliability

Internal consistency was adequate with Cronbach’s α of .73, .79, and .85 for the Meaning, Peace, and Faith subscales, respectively. Cronbach’s α for the Total scale was .79. Mean inter-item correlation was .54. Table 3 reports means, standard deviations and reliabilities for FACIT-Sp scores.

**Table 3:** FACIT-Sp mean scores and reliabilities

	*M*	*SD*	Min	Max	Cronbach’s *α*
Meaning	8.79	2.77	2	15	.73
Peace	6.25	2.15	1	14	.79
Faith	7.35	4.61	0	16	.85
Total	22.84	7.00	6	42	.79

### Literature review

**Fig. 1:**
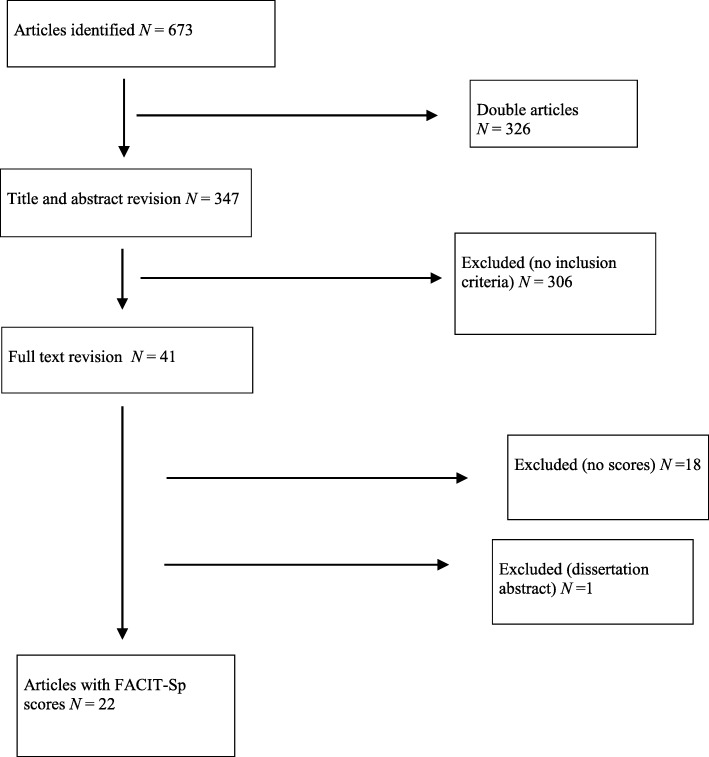
Flow-chart of the review

The literature review yielded 44 studies. Of these, 22 were excluded because they did not meet the inclusion criteria. Finally, 22 articles were included in the review (see Fig. 1).

The 22 articles that are included in the review are summarized in Table S2.

#### Reference intervals

Papers were classified according to the following categories:

1. newly diagnosed cancer patients [3, 24, 33, 34];
2. cancer survivors [35–38];
3. terminal cancer patients [39–42].

Terminal cancer patients had the lowest scores on the Faith and Meaning dimensions, whereas cancer survivors had the highest score on the Faith dimension. Newly diagnosed cancer patients and cancer survivors had similar and the highest scores on the Meaning dimension, whereas the latter had the highest score on the Peace dimension. Table 4 reports the lower and upper limits for reference intervals.

**Table 4:** Reference intervals of FACIT-Sp scores

		Newly diagnosed cancer patients ^a^	Cancer Survivors	Terminal cancer patients ^a^
	n	205	35,179	85
	Lower	0.0	3.3	0.0
**Faith**	Mean	7.3	11.8	9.7
	Upper	15.6	20.1	20.8
	n	205	35,179	NA
	Lower	8.3	8.2	
**Meaning**	Mean	14	13.7	
	Upper	19.7	19.2	
	n	205	35,179	NA
	Lower	3.1	5.5	
**Peace**	Mean	10.2	12	
	Upper	17.3	18.5	
**Spiritual Well Being**	n	1272	NA	298
	Lower	15.9		3
	Mean	32.9		22.6
	Upper	50		42

^a^: Means and limits of Faith, Meaning and Peace for newly diagnosed cancer patients were based on [24, 33] only, as [3] calculated such scores using different items and [34] didn’t provide this information. Conversely, all papers related to this group were used to calculate means and limits of Spiritual Well Being as the total score includes all the items. Only [42] was used to calculate means and limits of Faith for terminal cancer patients, while means and limits of Spiritual Well Being for this group were calculated using all papers

## Discussion

Exploratory factor analysis confirmed the three-factor structure of the FACIT-Sp found in previous research [3, 21, 38, 43]. Indeed, different from the original two-factor solution [23], this structure reflects the conceptual difference between meaning and peace: the first reflecting a cognitive dimension, and the latter an affective dimension of religious and spiritual well-being [21, 38]. However, different from previous studies in which item 12 (“I know that whatever happens with my illness, things will be okay”) was located in the Peace factor [21, 43], in our study it was found to be located in the Meaning factor. Other studies [38, 44] have found a double loading of the item 12 on both Peace and Faith factors. The different factor loading for this item may reflect cultural differences; patients in our sample may have relied on meaning, rather than on peace or faith, as a coping mechanism used to make sense of their life despite the illness. Consistent with previous studies, Faith was moderately correlated with both meaning and peace, whereas the association between peace and meaning was medium to large.

In our review of studies using the FACIT-Sp, terminal cancer patients had the lowest scores on most subscales of the FACIT-Sp, indicating greater impairment in the spiritual well-being dimensions. This result seems somewhat unexpected, given that previous studies showed that awareness of terminal illness was associated with better spiritual well-being in terminal cancer patients [45]. However, comparisons with Leung et al.’s [45] findings are difficult because they used a different questionnaire to assess spiritual well-being. Moreover, we do not know if terminal cancer patients included in our review were aware of their cancer diagnosis and prognosis. Indeed, prognosis of a terminally ill condition is frequently not disclosed to maintain hope for patients and their families.

This study has some limitations. First, we collected data using a convenience sampling method. Therefore our results cannot be generalized to all cancer patients in Italy. Further studies with random sampling procedures are needed. Second, an examination of the FACIT-Sp concurrent validity is needed by using well-validated measures of spiritual well-being. Third, only advanced and terminally ill cancer patients took part in the study. Therefore, the generalization of our findings to different cancer patients requires caution. Further studies with different types of clinical groups (e.g., newly diagnosed cancer patients and cancer survivors) are needed to cross-validate our findings. The use of a multi-sample confirmatory factor analysis might be a useful approach [46]. Notwithstanding these limitations, this is the first study that examined the factorial validity of the FACIT-Sp with patients with advanced and terminally ill cancer who were also no newly diagnosed.

## Conclusions

There is a growing incidence of cancer worldwide [6], and meeting the spiritual needs of patients is a vital aspect of care [1]. Patients with serious illness and end-of-life issues have the desire to include spirituality in their care [18]. Indeed, spirituality can be an inner resource in helping patients find a new meaning in their existence by reevaluating their experience of illness, and recognize what ultimately matters most to them [11]. It is therefore essential that clinicians address regular assessment of patients’ spiritual issues, treat spiritual distress and promote a sense of meaning in life, purpose, and peacefulness as parts of a biopsychosocial-spiritual approach to end-of-life care.

The results of the present study confirmed the three-factor structure of the FACIT-Sp also in an Italian sample of terminally ill cancer patients who were also no newly diagnosed. To our knowledge, no previous studies have examined the psychometric properties of this instrument with these patients. Therefore, the FACIT-Sp is a valid and reliable instrument to measure spiritual well-being in these patients and to identify their spiritual strengths that may be essential for a person-centered care.

## Supplementary information

**Additional file 1: Table S1.** A comparison of the factor loadings of the FACIT-Sp scale by studies. **Table S2.** Papers included in the review [47–58].

## Data Availability

The datasets used and analysed during the current study are available from the corresponding author on reasonable request.

## Abbreviations

ECOG: Eastern Cooperative Oncology Group
FACIT-Sp: Functional Assessment of Chronic Illness Therapy-Spiritual
POS: Palliative Care Outcome Scale
QOL: Quality of Life
