# Supplementary material for: The assessment of spiritual well-being in cancer patients with advanced disease: which are its meaningful dimensions?
Source: BMC Palliat Care. 2020 Mar 6;19:26. doi: 10.1186/s12904-020-0534-2 (PMC7059276; doi:10.1186/s12904-020-0534-2)
Supplement: Supplementary file 1 — Additional file 1: Table S1. A comparison of the factor loadings of the FACIT-Sp scale by studies. Table S2. Papers included in the review [47–58]. [file 12904_2020_534_MOESM1_ESM.doc]

**Table S1. A comparison of the factor loadings of the FACIT-Sp scale by studies**

| Model | No. Factors | Population | *n* | Extraction method | Allocation of items to scales | | |
| --- | --- | --- | --- | --- | --- | --- | --- |
|  |  |  |  |  | Peace | Faith | Meaning |
| Peterman et al. 2002 [23] | 2 | Cancer or HIV/AIDS | 1617 | PCA | 1,2,3,4,5*,6*,7*,8 | 9,10,11,12* |  |
| Noguchi et al. 2004** [47] | 2 | Cancer | 306 | EFA | 1,2,3,4,5,6,7,8 | 9,10,11,12 |  |
| Canada et al. 2008 [21] | 3 | Cancer | 240 | CFA | 1,4,6,7 | 9,10,11,12 | 2,3,5,8 |
|  |  |  |  | EFA | 1,4,6,7,8* | 9,10,11,12 | 2,3,5 |
| Murphy et al. 2010 [38] | 3 | Cancer | 8805 | CFA | 1,4,6,7 | 9,10,11,12* | 2,3,5,8 |
| Jafari et al. 2013b [48] | 3 | Cancer | 153 | CFA | 1,6,7 | 9,10,11,12 | 2,3,5 |
| Lazenby et al. 2013 [43] | 3 | Cancer | 205 | PAF | 1*,4,6*,7* | 9*,10,11,12* | 2,3,5*,8 |
| Bai & Dixon 2014 [24] | 3 | Cancer | 153 | PCA, IPFA, ML | 1,6,7,12 | 9,10,11 | 2,3*,4,5,8 |
| Haugan 2015 [44] | 3 | Nursing home | 202 | PCA | 1,4,8* | 9,10,11,12 | 2,3,5,6,7* |
|  |  |  |  | CFA | 1,4,6,7 | 9,10,11,12* | 2,3,5,8 |
| Agli et al. 2017 [49] | 3 | Nursing home | 63 | CFA | 1,4,6,7 | 9,10,11 | 2,5,8,12 |
| Akturk et al. 2017 [50] | 3 | Cancer | 137 | PCA | 1,4,6,7 | 9,10,11,12 | 2,3,5,8 |
| § Rabitti et al. 2020 | 3 | Cancer | 150 | PAF | 1,4,6,7 | 9,10,11 | 2,3,5,8,12 |
| *Note*. PCA = principal component analysis; EFA = exploratory factor analysis; CFA = confirmatory factor analysis; PAF = principal axis factoring; IPFA = iterative principal factor analysis; ML = maximum likelihood.  * Items with cross-loadings (>.30) on two factors  ** All items with cross-loadings >.30 on two factors  § This article refers to the present study and is not included in the reference list | | | | | | | |

**Table S2. Papers included in the review.**

| Article | N | Patients | FACIT-Sp scores: *M* (*SD*) |
| --- | --- | --- | --- |
| Schubart et al, 2010 [51] | 93 | Gastro Intestinal Cancer. Follow up patients (they had completed their surgical and adjuvant therapies). | **Total:**  Spiritual well being *M* = 38.5 (7.5)  **Pancreas (*N* = 37)**  Spiritual well being *M* = 39.1  **Liver (*N* = 32)**  Spiritual well being *M* = 37.7  **Esophagus and gastric (*N* = 16)**  Spiritual well being *M* = 36.3  **Sarcoma (*N* = 11)**  Spiritual well being *M* = 41.5 |
| Ando et al. 2010 [40] | 68 | Terminally ill cancer patients | Spiritual well being *M* = 16.7 (8.6) |
| Cook & Silverman, 2013 [52] | 17 | Inpatients at an oncology-hematology unit | **Control group post-test (*N* = 7)**  FACIT-Sp Meaning *M* = 13.6 (0.83)  FACIT-Sp Peace *M* = 12.1 (0.6)  FACIT-Sp Faith *M* = 12.3 (0.7)  Spiritual well being *M* = 39.9 (1.1) |
| Piderman et al. 2014 [53] | 64 | Patients who had been diagnosed with advanced stage cancer within the past year and were receiving radiation therapy. They had a life expectancy of greater than 6 month but less than 5 years. | FACIT-Sp Meaning/Peace *M* = 82.8 (16.2)  FACIT-Sp Faith *M* = 80.2 (22.0)  Spiritual well being *M* = 81.9 (15.4) |
| Sun et al. 2015 [54] | 475 | Patients with a diagnosis of stage I-IV non small cell lung cancer | FACIT-Sp Meaning/Peace *M* = 25.8 (5.6)  FACIT-Sp Faith *M* = 10.9 (4.9)  Spiritual well being *M* = 36.7 (8.6) |
| Jafari et al. 2013a [34] | 68 | Newly diagnosed breast cancer patients under radiation therapy | FACIT-Sp Meaning *M* = 9.8 (3.1)  FACIT-Sp Peace *M* = 7.2 (2.9)  FACIT-Sp Faith *M* = 11.3 (3.0)  Spiritual well being *M* = 28.4 (6.9) |
| Gonzalez et al. 2014 [55] | 98 | Patients with different kind of cancer and different stages | FACIT-Sp Meaning/Peace *M* = 24.3 (5.9)  FACIT-Sp Faith *M* = 11.9 (4.5)  Spiritual well being *M* = 36.2 (9.4) |
| Canada et al. 2013 [35] | 9105 | Cancer survivors | **FACIT-Sp Meaning:**  **All *M=*13.7 (2.8)**  White *M* = 13.7 (2.8)  Black *M* = 13.7 (2.8)  Hispanic *M* = 13.5 (2.9)  Years < 60 *M* = 13.6 (3.0)  Years 60-74 *M* = 14.0 (2.6)  Years > 75 *M* = 13.3 (2.9)  Female *M* = 13.7 (2.8)  Male *M* = 13.6 (2.8)  Married *M* = 13.9 (2.6)  Not married *M* = 13.0 (3.2)  Less than high school *M* = 12.9 (3.1)  High school *M* = 13.5 (2.9)  Some college *M*=13.7 (2.9)  Bachelor’s degree/Postgraduate degree *M* = 14.1 (2.5)  Breast cancer *M* = 13.7 (2.8)  Prostate cancer *M* = 13.7 (2.8)  Colorectal cancer *M* = 13.6 (2.8)  Bladder cancer *M* = 13.4 (2.9)  Uterine cancer *M* = 13.7 (32.9)  Melanoma *M* = 13.8 (2.7)  Time since diagnosis 2 years *M* = 13.7 (2.9)  Time since diagnosis 5 years *M* = 13.7 (2.8)  Time since diagnosis 10 years *M* = 13.6 (2.8)  None comorbid conditions *M* = 14.4 (2.3)  One comorbid conditions *M* = 14.1 (2.5)  Two comorbid conditions *M* = 13.7 (2.7)  Three or + comorbid conditions *M* = 12.7 (23.3)  **FACIT-Sp Peace:**  **All *M*= 12.0 (3.3)**  White *M* = 11.9 (3.4)  Black *M* = 12.5 (3.4)  Hispanic *M* = 11.8 (3.4)  Years < 60 *M* = 11.3 (3.7)  Years 60-74 *M* = 12.3 (3.3)  Years > 75 *M* = 12.1 (3.3)  Female *M* = 11.9 (3.5)  Male *M* = 12.1 (3.3)  Married *M* = 12.1 (3.3)  Not married *M* = 11.6 (3.6)  Less than high school *M* = 11.4 (3.5)  High school *M* = 11.8 (3.5)  Some college *M* = 12.0 (3.4)  Bachelor’s degree/Postgraduate degree *M* = 12.4 (3.2)  Breast cancer *M* = 11.8 (3.5)  Prostate cancer *M* = 12.2 (3.3)  Colorectal cancer *M* = 12.1 (3.4)  Bladder cancer *M* = 11.8 (3.6)  Uterine cancer *M* = 12.1 (3.6)  Melanoma *M* = 11.8 (3.3)  Time since diagnosis 2 years *M* = 11.9 (3.5)  Time since diagnosis 5 years *M* = 12.0 (3.4)  Time since diagnosis 10 years *M* = 12.0 (3.3)  None comorbid conditions *M* = 12.9 (3.0)  One comorbid conditions *M* = 12.4 (3.1)  Two comorbid conditions *M* = 12.1 (3.3)  Three or + comorbid conditions *M* = 10.8 (3.8)  **FACIT-Sp Faith:**  **All *M* = 11.8 (4.3)**  White *M* = 11.3 (4.4)  Black *M* = 13.8 (3.3)  Hispanic *M* = 12.6 (3.9)  Years < 60 *M* = 11.3 (4.5)  Years 60-74 *M* = 11.9 (4.2)  Years > 75 *M* = 11.7 (4.4)  Female *M* = 12.3 (4.2)  Male *M* = 11.0 (4.5)  Married *M* = 11.7 (4.3)  Not married *M* = 11.8 (4.4)  Less than high school *M* = 12.5 (4.1)  High school *M* = 12.1 (4.1)  Some college *M* = 11.8 (4.3)  College + *M* = 11.0 (4.6)  Breast cancer *M* = 12.3 (4.1)  Prostate cancer *M* = 11.1 (4.4)  Colorectal cancer *M* = 11.9 (4.4)  Bladder cancer *M* = 11.0 (4.6)  Uterine cancer *M* = 12.2 (4.1)  Melanoma *M* = 10.6 (4.4)  Time since diagnosis 2 years *M* = 11.6 (4.3)  Time since diagnosis 5 years *M* = 11.7 (4.3)  Time since diagnosis 10 years *M* = 11.8 (4.3)  None comorbid conditions *M* = 11.8 (4.3)  One comorbid conditions *M* = 11.9 (4.3)  Two comorbid conditions *M* = 11.8 (4.3)  Three or + comorbid conditions *M* = 11.4 (4.4) |
| Bai et al. 2016 [33] | 52 | Patients newly diagnosed with advanced cancer (stage III or IV) diagnosed as head, neck and gastrointestinal cancer undergoing treatment. | FACIT-Sp Meaning *M* = 17.3 (3.2)  FACIT-Sp Peace *M* = 11.9 (3.1)  FACIT-Sp Faith *M* = 7.2 (4.3)  Spiritual well being *M* = 36.7 (8.2) |
| Canada et al. 2016 [36] | 8405 | Patients with stage I-IV cancer diagnosed 2, 5 or 10 years prior to the time of sampling. | FACIT-Sp Meaning *M* = 13.7 (2.8)  FACIT-Sp Peace *M* = 12.0 (3.4)  FACIT-Sp Faith *M* = 11.7 (4.3)  Spiritual well being *M* = 37.3 (8.6) |
| Munoz et al. 2015 [37] | 8864 | Patients diagnosed with prostate, breast, colorectal, bladder, uterine or skin melanoma cancer. Diagnosed 2, 5 or 10 years before sampling. | FACIT-Sp Meaning *M* = 13.67 (2.82)  FACIT-Sp Peace *M* = 11.99 (3.42)  FACIT-Sp Faith *M* = 11.70 (4.35)  FACIT-Sp Meaning\peace *M* = 25.65 (5.74)  Spiritual well being *M* = 37.35 (8.65) |
| Bovero et al. 2016 [41] | 115 | Patients diagnosed with cancer and a life expectancy of 4 month or less and Karnosfky Performance Status ≤ 40 | **Patients with low quality of life (measured by FACIT-G):**  Spiritual well being *M* = 16.7 (8.1)  **Patients with high quality of life (measured by FACIT-G):**  Spiritual well being *M* = 26.7 (9.1) |
| Lazenby & Khatib, 2012 [56] | 159 | Inpatients and outpatients in treatment for cancer, aware of their illness | **Breast cancer:**  FACIT-Sp Meaning *M* = 9.8 (2.7)  FACIT-Sp Peace *M* = 9.1 (2.7)  FACIT-Sp Faith *M* = 13.8 (6.5)  Spiritual well being *M* = 32.90 (6.6)  **Bone/Sarcoma:**  FACIT-Sp Meaning *M* = 9.9 (1.9)  FACIT-Sp Peace *M* = 10.5 (1.6)  FACIT-Sp Faith *M* = 15.4 (0.8)  Spiritual well being *M* = 35.7 (3.6)  **Gastrointestinal Cancer:**  FACIT-Sp Meaning *M* = 10.3 (2.1)  FACIT-Sp Peace *M* = 10.1 (2.1)  FACIT-Sp Faith *M* = 14.7 (1.9)  Spiritual well being *M* = 35.1 (4.5)  **Gynecologic Cancer:**  FACIT-Sp Meaning *M* = 10.1 (1.8)  FACIT-Sp Peace *M* = 9.7 (2.1)  FACIT-Sp Faith *M* = 14.3 (1.7)  Spiritual well being *M* = 34.1 (4.5)  **Lung Cancer:**  FACIT-Sp Meaning *M* = 12.5 (1.5)  FACIT-Sp Peace *M* = 10.8 (2.6)  FACIT-Sp Faith *M* = 14.9 (1.3)  Spiritual well being *M* = 38.3 (4.0)  **Other:**  FACIT-Sp Meaning *M* = 10.4 (2.1)  FACIT-Sp Peace *M* = 10.0 (2.2)  FACIT-Sp Faith *M* = 14.1 (2.3)  Spiritual well being *M* = 34.5 (4.7) |
| Rodin et al. 2007 [57] | 326 | Outpatients diagnosed with stage IIIA, IIIB or IV lung cancer or stage IV gastrointestinal cancer. | FACIT-Sp Meaning/Peace *M* = 3.2 (0.7)  FACIT-Sp Faith *M* = 2.4 (1.2) |
| Peterman et al. 2002 [23] | 1617  (First study)  131  (Second Study) | 83.1% of patients had cancer (other patients had HIV\AIDS)  Sample of patients beginning chemotherapy for any solid tumor or haematological malignancy. | FACIT-Sp Meaning/Peace *M* = 25.2 (5.6)  FACIT-Sp Faith *M* = 13.3 (3.6)  Spiritual well being *M* = 38.5 (8.1)  **Breast cancer**  FACIT-Sp Meaning/Peace *M* = 26.1  FACIT-Sp Faith *M* = 13.8  Spiritual well being *M* = 39.9  **Colorectal cancer**  FACIT-Sp Meaning/Peace *M* = 26.1  FACIT-Sp Faith *M* = 14.0  Spiritual well being *M* = 40.2  **Head and neck cancer**  FACIT-Sp Meaning/Peace *M* = 25.2  FACIT-Sp Faith *M* = 13.6  Spiritual well being *M* = 38.8  **Lung cancer**  FACIT-Sp Meaning/Peace *M* = 25.3  FACIT-Sp Faith *M* = 13.2  Spiritual well being *M* = 38.5  FACIT-Sp Meaning/Peace *M* = 25.0 (5.4)  FACIT-Sp Faith *M* = 11.8 (4.3)  Spiritual well being *M* = 36.8 (8.3) |
| Wang et al. 2016 [42] | 85 | Patients with terminal cancer | FACIT-Sp Meaning *M* = 21.3 (8.8)  FACIT-Sp Faith *M* = 9.7 (5.6)  Spiritual well being *M* = 31.0 (12.9) |
| Bai & Dixon 2014 [24] | 153 | Patients diagnosed with advanced cancer | FACIT-Sp Meaning *M* = 16.7 (3.4)  FACIT-Sp Peace *M* = 11.3 (3.6)  FACIT-Sp Faith *M* = 7.4 (4.2) |
| Ando et al. 2008 [39] | 30 | Patients with incurable cancer | Spiritual well being *M* = 16.0 (8.2) |
| Wittmann et al. 2006 [58] | 88 | Inpatients with haematological malignancies | Spiritual well being *M* = 32.3 (7.5) |
| Jafari et al. 2013b [48] | 153 | Patients with malignancies (stage I, II, III, IV) | Spiritual well being *M* = 32.6 (6.4) |
| Whitford & Olver, 2012 [3] | 999 | Newly diagnosed cancer patients | FACIT-Sp Meaning *M* = 13.7 (2.8)  FACIT-Sp Peace *M* = 10.1 (3.7)  FACIT-Sp Faith *M* = 8.4 (4.9)  Spiritual well being *M* = 33.0 (9.0) |
| Murphy et al. 2010 [38] | 8805 | Cancer survivors (stage I, II, III, IV) | FACIT-Sp Meaning *M* = 13.7 (2.8)  FACIT-Sp Peace *M* = 12.0 (3.4)  FACIT-Sp Faith *M* = 11.7 (4.3)  Spiritual well being *M* = 37.3 (8.6) |

*Note*. We decided to report only the most relevant data for our descriptive purposes.
